# In Vivo Zymosan Treatment Induces IL15-Secreting Macrophages and KLRG1-Expressing NK Cells in Mice

**DOI:** 10.3390/molecules28155779

**Published:** 2023-07-31

**Authors:** Hyun Jung Park, Sung Won Lee, Yun Hoo Park, Tae-Cheol Kim, Sujin Lee, Seyeong Lee, Luc Van Kaer, Seokmann Hong

**Affiliations:** 1 Department of Integrative Bioscience and Biotechnology, Institute of Anticancer Medicine Development, Sejong University, Seoul 05006, Republic of Korea; 0402parkhj@gmail.com (H.J.P.); dbsgn703@gmail.com (Y.H.P.); mitalus1@gmail.com (T.-C.K.); lsj1262487@gmail.com (S.L.); lsyjhs0218@gmail.com (S.L.); 2 Department of Biomedical Laboratory Science, College of Health and Biomedical Services, Sangji University, Wonju 26339, Republic of Korea; sungwonlee@sangji.ac.kr; 3 Department of Pathology, Microbiology and Immunology, Vanderbilt University School of Medicine, Nashville, TN 37232, USA; luc.van.kaer@vumc.org

**Keywords:** beta-glucan, zymosan, pustulan, natural killer cells, macrophages

## Abstract

Beta-glucan (β-glucan) is a natural polysaccharide produced by fungi, bacteria, and plants. Although it has been reported that β-glucan enhances innate immune memory responses, it is unclear whether different types of β-glucans display similar immune effects. To address this issue, we employed zymosan (β-1,3-glycosidic linkage) and pustulan (β-1,6-glycosidic linkage) to investigate their in vivo effects on innate memory immune responses. We examined the changes of innate memory-related markers in macrophages and natural killer (NK) cells, two immune cell types that display innate memory characteristics, at two different time points (16 h and 7 days) after β-glucan stimulation. We found that short-term (16 h) zymosan treatment significantly induced macrophages to upregulate IL15 production and increased surface IL15Rα expression on NK cells. In addition, long-term (7 days) zymosan treatment significantly induced macrophages to upregulate the expression of innate memory-related markers (e.g., TNFα, HIF1α, and mTOR) and induced NK cells to express enhanced levels of KLRG1, known as an innate memory-like marker. Our results provide support that zymosan can be an effective adjuvant to promote innate memory immune responses, providing a bridge between innate and adaptive immune cells to enhance various immune responses such as those directed against tumors.

## 1. Introduction

Beta-glucan (β-glucan) is a major component of the microbial cell wall (e.g., bacteria, archaea, and fungi) and is composed of a linear polymer of β–d-glucose polysaccharides linked by either β-1,3- or β-1,6-glycosidic bonds [1]. Upon recognition by C-type lectin receptors, such as dectin-1 and toll-like receptor 2 (TLR2) expressed on innate immune cells (i.e., macrophages and dendritic cells (DCs)) [1], β-glucan can activate these cells to produce inflammatory cytokines [2]. It has been reported that macrophages preactivated by β-glucan treatment display more enhanced and persistent innate immune status after subsequent recognition of pathogen-associated molecular patterns (PAMPs) (i.e., LPS), which is mediated via epigenetic reprogramming. A more robust innate immune response to downstream infections is referred to as an innate memory immune response (also termed trained immunity) [3,4]. For example, *Candida albicans*-derived β-glucan linked by β-1,3-glycosidic bonds promotes trained immunity of macrophages via epigenetic reprogramming [5]. On the other hand, pustulan obtained from the cell wall of *Lasallia pustulata* contains β-1,6-glycosidic linkages and induces an anti-inflammatory effect via a macrophage-dependent pathway [6].

Zymosan is a β-1,3-glycosidic-linked β-glucan derived from *Saccharomyces cerevisiae.* Like other β-glucans, zymosan can activate innate immune cells such as DCs and macrophages to express reactive oxygen species (ROS) and proinflammatory cytokines such as TNFα [7]. However, one recent study demonstrated that intraocular injection of zymosan induces central nervous system neuron survival and axon regeneration by accumulating anti-inflammatory neutrophils in the optic nerve crush injury site [8]. Thus, the role of zymosan in immune cell activation has yet to be fully elucidated.

Natural killer (NK) cells are one of the innate immune cells that play essential roles in antiviral immunity. Despite their innate immune properties, NK cells can display adaptive immune characteristics, such as memory development, at least to some antigenic stimuli and under certain experimental conditions. For example, NK cells can elicit memory-like immune responses in situations such as viral infection with mouse cytomegalovirus (MCMV). MCMV-activated NK cells express higher levels of KLRG1, known as a memory-like marker, compared with unstimulated naive NK cells [9]. In addition, the differentiation of KLRG1^+^ NK cells is known to be induced by interleukin-15 (IL15), and these NK cells contribute to antitumor immunity against pulmonary metastatic colorectal carcinoma [10]. KLRG1-expressing mature NK cells accumulate upon sustained but not transient stimulation with recombinant IL15/IL15α complex treatment, implying that these cells might be preferentially differentiated under chronic inflammatory conditions [11]. KLRG1 expression and effector functions of NK cells require hypoxia-inducible factor-1α (HIF1α) during MCMV infection [12].

In this study, we investigated zymosan’s in vivo immunological effects compared with 1,6-linked pustulan, focusing on the innate memory immune responses mainly in macrophages and NK cells. For this purpose, we examined β-glucan’s effect on innate immune phenotypes of macrophages and NK cells (compared with NKT cells, innate-like T cells with similar cytotoxic capacities to NK cells) at two different time points (16 h and seven days) post-stimulation.

## 2. Results

### 2.1. In Vivo Zymosan Treatment Activates Splenic Macrophages to Produce High Levels of IL15

**Figure 1:**
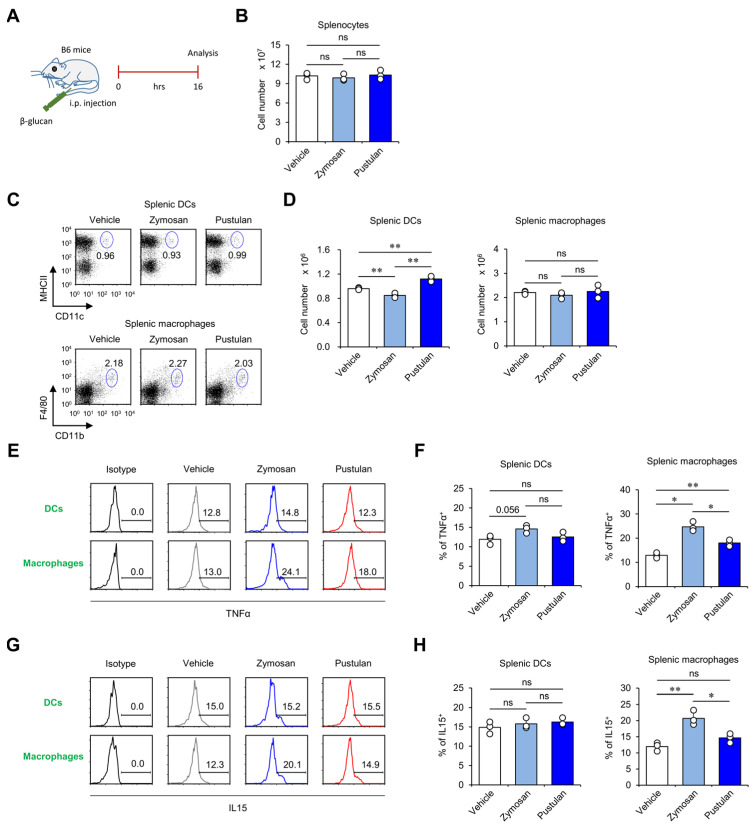
In vivo zymosan treatment activates splenic macrophages to produce high levels of IL15. (**A**–**G**) WT mice were intraperitoneally (i.p.) injected with either zymosan or pustulan. Splenocytes were harvested from these mice at 16 h post-injection. (**B**) Total splenocyte cell numbers were measured. (**C**,**D**) The frequency and absolute numbers of DCs (MHCII^+^CD11c^+^) and macrophages (CD11b^+^F4/80^+^CD11c^−^) were determined by flow cytometry. (**C**) Representative FACS plots; (**D**) summary figures. Production of (**E**,**F**) TNFα and (**G**,**H**) IL15 in DCs and macrophages was assessed via flow cytometry. (**E**,**G**) Representative FACS histograms; (**F**,**H**) summary figures. The mean values ± SD (*n* = 3 per group in the experiment; Student’s *t*-test; * *p* < 0.05, ** *p* < 0.01) are shown. One representative experiment of two experiments is shown. ns: not significant.

To examine the in vivo effect of β-glucans, two different β-glucans, zymosan and pustulan, were employed. It has been reported that zymosan with β-1,3-linkages shows immunostimulatory effects, whereas pustulan with β-1,6-linkages exhibits immunosuppressive effects [6,7]. However, the effects of β-glucans on innate immune responses have yet to be fully elucidated. To address this issue, wild-type (WT) C57BL/6 (B6) mice were intraperitoneally (i.p.) injected with either zymosan or pustulan, and the extent of activation (i.e., cell numbers and cytokine production) of DCs and macrophages was examined in the spleen between vehicle (veh)- and β-glucan-injected mice (Figure 1A,B). In vivo zymosan or pustulan treatment slightly but significantly decreased or increased the total cell numbers of DCs, respectively, whereas both β-glucans failed to alter the cell numbers of splenic macrophages (Figure 1C,D).

Next, we examined whether there is any qualitative change(s) in DCs or macrophages after β-glucan injection. For this purpose, we evaluated cytokine production by these cells upon β-glucan stimulation. Intriguingly, we found that the frequency of TNFα- and IL15-secreting macrophages but not DCs was increased at 16 h post-treatment, indicating that both zymosan and pustulan possess immunostimulatory activities (Figure 1E–H). In addition, the frequency of IL15-secreting macrophages was increased in zymosan-injected mice compared with pustulan-injected mice (Figure 1G,H). These results suggest that zymosan is superior to pustulan in activating macrophages to produce IL15.

### 2.2. In Vivo Zymosan Treatment Induces the Activation of NK and NKT Cells

**Figure 2:**
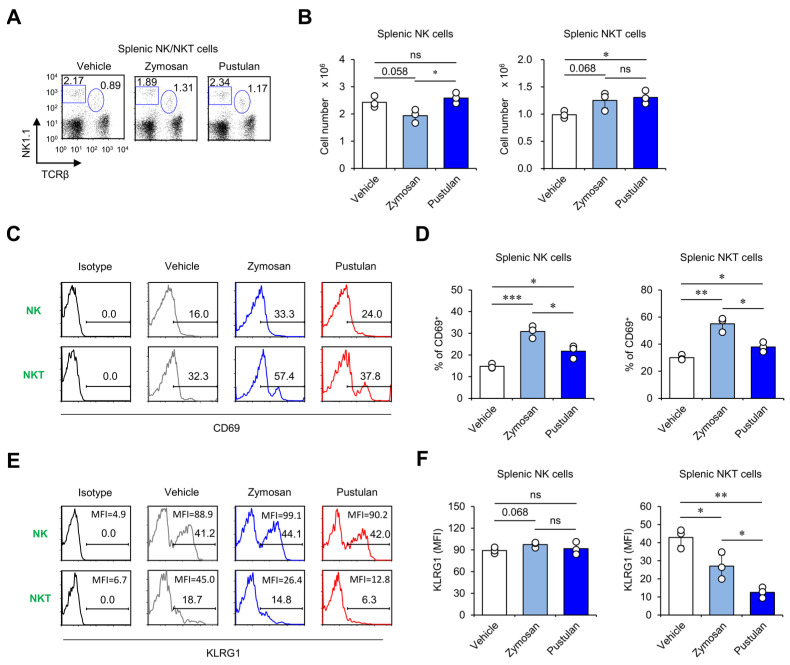
In vivo zymosan treatment induces the activation of NK and NKT cells. (**A–F**) WT mice were i.p. injected with zymosan or pustulan. Splenocytes were harvested from these mice at 16 h after injection. (**A**,**B**) The frequency and absolute numbers of NK (TCRβ^-^NK1.1^+^) and NKT cells (TCRβ^+^NK1.1^+^) were determined by flow cytometry. Surface expression of (**C**,**D**) CD69 and (**E**,**F**) KLRG1 in NK and NKT cells was assessed via flow cytometry. (**C**,**E**) Representative FACS histograms; (**D**,**F**) summary figures. The mean values ± SD (*n* = 3 per group in the experiment; Student’s *t*-test; * *p* < 0.05, ** *p* < 0.01, *** *p* < 0.001) are shown. One representative experiment of two experiments is shown. ns: not significant.

It has been reported that in vivo application of β-glucan induces IFNγ production by leukocytes in mice [13]. Because NK and NKT cells provide an early source of IFNγ production in the spleen, as shown previously [14,15], we examined whether in vivo injection of β-glucans (zymosan and pustulan) impacts these cells at 16 h post-treatment. Interestingly, zymosan treatment appeared to affect the cellularity of both NK and NKT cells: there was a tendency for a decrease in NK cells (*p* = 0.058) and an increase in NKT cells (*p* = 0.068) at 16 h after treatment (Figure 2A,B). However, pustulan appeared to increase NKT cells but not NK cells (Figure 2A,B). Next, we examined the effect of zymosan and pustulan on the activation of NK and NKT cells. As expected, both zymosan and pustulan efficiently increased the expression of early activation markers (e.g., CD69) in these cells at 16 h after treatment (Figure 2C,D). It has been previously reported that IL15 promotes the survival of NK cells by preventing apoptosis and upregulating the innate memory-like marker KLRG1 [11,16]. Since our results showed that macrophages from zymosan-injected mice secrete significantly raised levels of IL15, we analyzed the expression of KLRG1 on the surface of NK and NKT cells in both zymosan- and pustulan-injected mice. Zymosan but not pustulan tended to (but not significantly) increase KLRG1 expression on NK cells at 16 h after treatment. Surprisingly, both zymosan and pustulan injections decreased the prevalence of KLRG1-expressing NKT cells. Furthermore, pustulan’s effects were significantly more profound than zymosan’s effects (Figure 2E,F). These data indicate that NK cells can be activated by β-glucans but do not express KLRG1 at early time points (16 h post-stimulation).

### 2.3. In Vivo Zymosan Treatment Increases IL15 Receptor Expression on NK Cells

**Figure 3:**
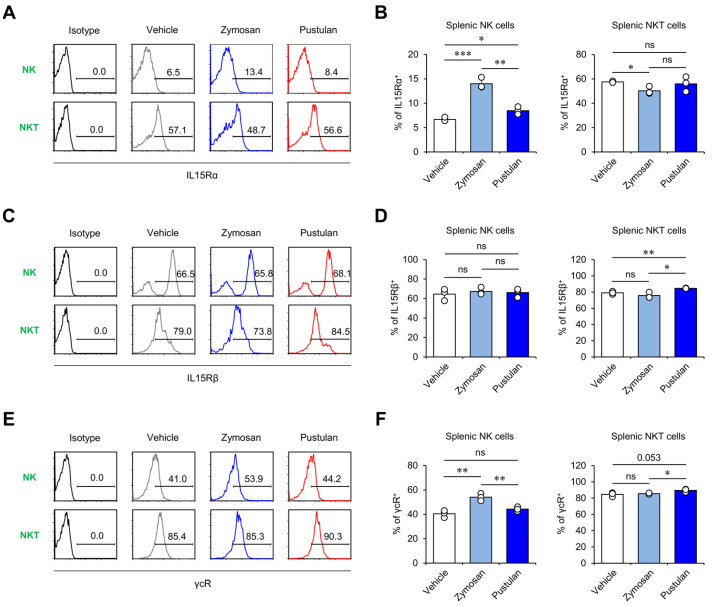
In vivo zymosan treatment increases IL15 receptor expression in NK cells. (**A**–**F**) WT mice were i.p. injected with zymosan or pustulan. Splenocytes were harvested from these mice at 16 h post-injection. Surface expression of (**A**,**B**) IL15Rα, (**C**,**D**) IL15Rβ, and (**E**,**F**) γcR in NK (TCRβ^-^NK1.1^+^) and NKT cells (TCRβ^+^NK1.1^+^) was determined by flow cytometry. (**A**,**C**,**E**) Representative FACS histograms; (**B**,**D**,**F**) summary figures. The mean values ± SD (*n* = 3 per group in the experiment; Student’s *t*-test; * *p* < 0.05, ** *p* < 0.01, *** *p* < 0.001) are shown. One representative experiment of two experiments is shown. ns: not significant.

Since zymosan can activate macrophages to produce IL15, which is known to play a role in stimulating NK and NKT cells, we next investigated whether β-glucan treatment can alter surface-level expression of the IL15 receptor (IL15R) on NK and NKT cells. IL15R consists of IL15Rα (also known as CD215), IL15Rβ (also known as IL2Rβ or CD122), and the common gamma chain (γc) (also known as CD132) [17]. To examine whether β-glucan treatment can modulate IL15R expression on NK and NKT cells, we compared the surface levels of IL15Rα, IL15Rβ, and γcR on these cells. We found that zymosan induced significantly higher levels of IL15Rα and γcR expression on NK cells but not NKT cells than veh or pustulan at 16 h after treatment (Figure 3A,B,E,F). In addition, in vivo treatment with either zymosan or pustulan failed to affect the expression of IL15Rβ on NK cells (Figure 3B). Interestingly, however, pustulan- but not zymosan-injected mice displayed a significant increase in IL15Rβ expression and a tendency toward increased γcR expression on NKT cells, indicating that pustulan may have unique effects on NKT cells (Figure 3C–F). These results indicate that zymosan and pustulan may have divergent effects on activating NK and NKT cells in vivo.

### 2.4. In Vivo Zymosan Treatment Upregulates Innate Memory-Related Markers on Macrophages

**Figure 4:**
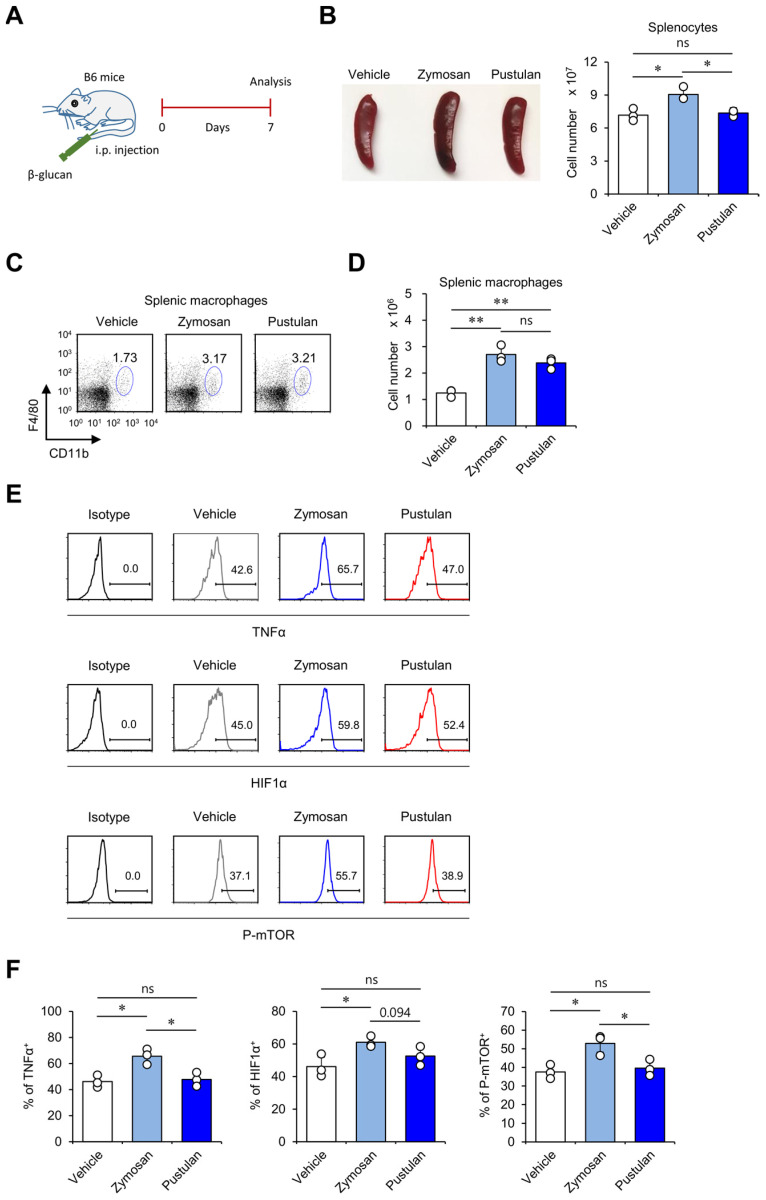
In vivo zymosan treatment upregulates innate memory-related markers on macrophages. (**A–F**) WT mice were i.p. injected with zymosan or pustulan. Splenocytes were harvested from these mice at seven days post-injection. (**B**) Splenocyte numbers were evaluated. (**C**,**D**) The frequency and absolute numbers of macrophages (CD11b^+^F4/80^+^CD11c^−^) were determined by flow cytometry. (**E**,**F**) Intracellular expression of TNFα, HIF1α, and P-mTOR in macrophages was assessed via flow cytometry. (**E**) Representative FACS histogram; (**F**) summary figures. The mean values ± SD (*n* = 3 per group in the experiment; Student’s *t*-test; * *p* < 0.05, ** *p* < 0.01) are shown. One representative experiment of two experiments is shown. ns: not significant.

To examine the effect of β-glucans on innate memory phenotypes of macrophages, we analyzed the cell numbers and innate memory markers of macrophages at day 7 after β-glucan treatment. First, we measured the cell numbers of splenocytes and found that zymosan- but not pustulan-injected mice displayed increased numbers of splenocytes compared with veh-injected mice (Figure 4A,B). Furthermore, we analyzed the absolute cell numbers of splenic macrophages and found that both zymosan and pustulan treatment induced a significant increase of macrophages in the spleen (Figure 4C,D). Since β-glucans are known to stimulate macrophages to induce innate memory-related signaling pathways, such as mechanistic Target of Rapamycin (mTOR) [2], we also examined the expression of TNFα, HIF1α, and P-mTOR in splenic macrophages at seven days post-β-glucan treatment. Interestingly, we found that macrophages from zymosan-injected mice expressed significantly higher levels of innate memory markers (TNFα, HIF1α, and P-mTOR) than veh- or pustulan-injected mice (Figure 4E,F). These results identify zymosan as a biomaterial that can promote innate memory-like phenotypes in macrophages.

### 2.5. In Vivo Zymosan Treatment Upregulates Expression of Memory Markers on NK Cells

**Figure 5:**
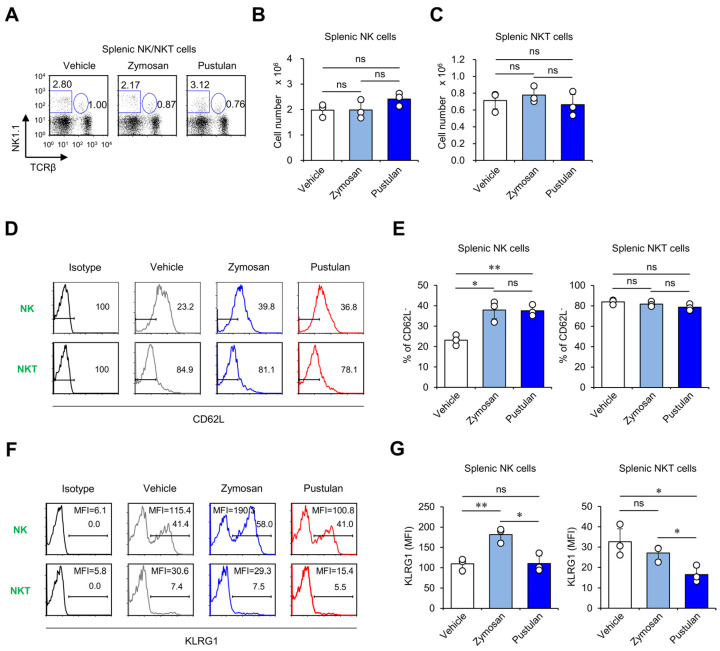
In vivo zymosan treatment upregulates expression of memory markers in NK cells. (**A–G**) WT mice were i.p. injected with zymosan or pustulan. Splenocytes were harvested from these mice at seven days post-injection. (**A**) The frequency and (**B**,**C**) absolute cell numbers of NK (TCRβ^-^NK1.1^+^) and NKT cells (TCRβ^+^NK1.1^+^) were determined by flow cytometry. Surface expression of (**D**,**E**) CD62L and (**F**,**G**) KLRG1 in NK and NKT cells was assessed via flow cytometry. (**D**,**F**) Representative FACS histograms; (**E**,**G**) summary figures. The mean values ± SD (*n* = 3 per group in the experiment; Student’s *t*-test; * *p* < 0.05, ** *p* < 0.01) are shown. One representative experiment of two experiments is shown. ns: not significant.

We next examined whether in vivo treatment of zymosan and pustulan can affect memory-like phenotypes of NK and NKT cells. For this purpose, we analyzed the expression of memory/effector markers (i.e., KLRG1 and CD62L) on NK and NKT cells at seven days after β-glucan injection. Although in vivo administration of zymosan and pustulan did not alter the total cell numbers of NK and NKT cells (Figure 5A–C), both β-glucans profoundly decreased the frequency of CD62L-expressing NK cells, whereas no effects of either β-glucan on CD62L expression by NKT cells was observed. Next, we evaluated whether β-glucan treatment can affect the expression of memory marker KLRG1 on NK and NKT cells. Intriguingly, only zymosan treatment significantly increased KLRG1 expression on NK cells at seven days after treatment, indicating that zymosan but not pustulan induces the differentiation of NK cells into memory-like NK cells (Figure 5D–G). However, the administration of zymosan and pustulan had little impact on the memory phenotype of NKT cells (Figure 5D–G), possibly due to their intrinsic memory properties. These results support the notion that in vivo zymosan treatment can induce NK cells displaying a memory-like phenotype.

## 3. Discussion

Here, we investigated whether in vivo treatment with two different β-glucans (zymosan and pustulan) can induce distinct innate memory phenotypes, particularly in NK cells. Whereas previous studies using β-glucans mainly focused on their effects on the memory phenotypes of monocytes and macrophages [5,18], it remains unclear whether β-glucans can affect the innate memory properties of NK cells.

Our results show that IL15 derived from macrophages correlates with the innate memory phenotype of NK cells in response to in vivo zymosan treatment. However, it is currently unknown whether the interaction of macrophages with NK cells requires direct engagement by receptor-ligand ligation to ultimately contribute to zymosan-mediated immune activation. Interestingly, it has been reported that polyriboinosinic-polyribocytidilic acid (poly I:C)-treated macrophages increased NK cell-mediated cytotoxicity against tumor cells through increased surface expression of RAE-1 (an NKG2D ligand) as well as production of IL15 [19]. In addition, the binding of NK cell receptor 2B4 to macrophage CD48 is essential for inducing NK cell proliferation and secretion of IFNγ following lipopolysaccharide (LPS) activation [20,21]. Moreover, such interactions are independent of NKp30, a critical molecule for DC-mediated NK cell activation [20,21]. Therefore, the engagement of either NKG2D-NKG2D ligand or 2B4-CD48 receptors may be responsible for crosstalk between macrophages and NK cells during zymosan-elicited proinflammatory immune responses. Thus, it will be worthwhile to further investigate the molecular mechanisms mediating the interactions between macrophages and NK cells.

Previous studies have reported that in vivo treatment with zymosan upregulates the expression of proinflammatory cytokines such as TNFα in peritoneal macrophages, ultimately inhibiting melanoma progression [22]. Moreover, zymosan repolarizes IL4-induced M2 macrophages towards an M1 phenotype producing TNFα and IL6 [23]. However, it has also been reported that pustulan possesses anti-inflammatory properties in the mannan-induced murine model of psoriatic arthritis, and such anti-inflammatory effects of pustulan are mediated by macrophages [6]. Consistent with previous studies, our results also demonstrate distinct effects of zymosan and pustulan on innate immune responses. In future studies, it will therefore be worthwhile to examine whether the distinct effects of zymosan and pustulan on KLRG1 expression by NK cells result from either linkage differences (1,3- or 1,6-linkage) or other unique features of these β-glucans.

Dysregulated IFNγ-mediated T helper 1 (Th1) responses are associated with a shift from balanced Th1/Th2 immune responses toward a Th2 cell-dominated profile in atopic dermatitis (AD) [24,25]. Our studies have shown that an increase in NKT cell-derived IFNγ inhibits the development of AD in NC/Nga mice by suppressing increased activation-induced cell death (AICD) of Th1 cells [26,27]. A numerical reduction and dysregulated IFNγ production by NK cells correlate with AD disease severity, indicating that NK cells might contribute to maintaining skin barrier functions at an early stage of AD [28]. NK cells from patients with AD exhibit susceptibility to AICD, but IL15 superagonism with rIL15/IL15Rα–Fc complexes promotes AD resolution in an NK cell-dependent manner, indicating that NK cells limit type 2 immune responses in AD [29]. Since our results showed that in vivo zymosan treatment significantly enhanced IL15 production by splenic macrophages (Figure 1), zymosan might prevent AD development by upregulating IFNγ-producing KLRG1^+^ NK cells, which will be explored in future studies.

Moreover, previous studies have reported that IL15 superagonist-stimulated NK cells enhance in vivo antitumor efficacy by promoting tumor infiltration [30], and IL15-expanded KLRG1^+^ NK cells protect mice from pulmonary metastatic colorectal carcinoma [10]. It is increasingly clear that β-glucans can enhance antitumor effects by stimulating immune cells, although they lack direct cytotoxic effects on tumor cells [31]. It will be interesting to examine whether the macrophage-IL15-NK cell axis elicited by zymosan treatment induces enhanced cytotoxicity against tumor cells in vitro and in vivo.

Interestingly, there are previous studies showing that IL6, known as one of the proinflammatory cytokines, has a distinct effect on NK cells compared with IL15. For example, tumor-derived IL6 inhibits the activity and function of NK cells [32]. IL6 suppresses the cytolytic activity of NK cells in the peritoneal fluid of patients with endometriosis, concomitantly downregulating NK cells’ cytolytic molecules (e.g., granzyme B and perforin) [33]. Since it has been known that macrophages produce IL6 upon zymosan treatment [34], it will be intriguing to investigate whether macrophage-derived IL6 induces similar or different effects on NK cells’ KLRG1 expression.

It has previously been demonstrated through many studies that β-glucans possess adjuvant activities on phagocytic cells (e.g., macrophages, monocytes, and DCs) during early immune responses [35,36]. In this study, our results provide evidence for the first time that in vivo treatment with zymosan increases macrophage-derived IL15 production, which may correlate with zymosan-induced upregulation of KLRG1 expression on NK cells. Taken together, our findings indicate that zymosan can be an excellent natural product that helps boost innate immune responses, bridging towards the generation of more effective antiviral or antitumor adaptive immunity.

## 4. Materials and Methods

### 4.1. Study Design

This study was designed to determine the effect of two different β-glucans on innate immune activation. To address this issue, WT B6 mice were intraperitoneally injected with β-glucan (zymosan or pustulan, 1 mg/mouse), and 16 h or 7 days later, splenocytes were harvested and further analyzed using flow cytometry. Sejong University Institutional review board approval was obtained before the experiment (SJ-20190301E1).

### 4.2. Mice and Reagents

WT B6 mice were purchased from Jung Ang Lab Animal Inc. (Seoul, Korea). These mice were maintained at Sejong University and were used for experiments at 6–12 weeks of age. The mice were maintained on a 12 h light/12 h dark cycle in a temperature-controlled barrier facility with free access to food and water. They were fed a γ-irradiated sterile diet and provided with autoclaved tap water. Age- and sex-matched male mice were used for all experiments. The animal experiments were approved by the Institutional Animal Care and Use Committee of Sejong University (SJ-20190301E1). Zymosan derived from *Saccharomyces cerevisiae* and pustulan derived from lichen *Lasallia pustulata* were purchased from InvivoGen (San Diego, CA, USA).

### 4.3. Flow Cytometry

The following monoclonal antibodies (mAbs) were obtained from BD Biosciences (San Jose, CA, USA): fluorescein isothiocyanate (FITC)- or phycoerythrin (PE)-Cy7-conjugated anti-TCRβ (clone H57-597); PE-Cy7- or allophycocyanin (APC)-conjugated ant-CD11c (clone HL3); PE- or APC-conjugated anti-NK1.1 (clone PK-136); PE-Cy7-conjugated CD69 (clone H1.2.F3); PE-Cy7-conjugated anti-CD11b (clone M1/70); PE-conjugated anti-TNFα (clone XP6-XT22); and PE-conjugated immunoglobulin G (IgG) (isotype control) (clone R3-34). In addition, the following mAbs from Thermo Fisher Scientific (Waltham, MA, USA) were used: FITC-, PE-Cy7-, or APC-conjugated anti-MHCII (clone M5/114.15.2); FITC- or APC-conjugated anti-Ly-6G (Gr-1) (clone RB6-8C5); APC-conjugated anti-F4/80 (clone BM8); PE-conjugated anti-KLRG1 (clone 2F1); PE-conjugate anti-CD62L (clone MEL-14); PE-conjugated anti-IL15Rα (clone DNT15Ra); and PE-conjugated anti-Phospho-mTOR (Ser2448) (clone MRRBY). The following mAbs from R&D systems (Minneapolis, MN, USA) were used: PE-conjugated anti-HIF1α (clone 241812). The following mAbs from BioLegend (San Diego, CA, USA) were used: PE-conjugated anti-IL2Rβ (clone 5H4); and PE-conjugated anti-CD132 (common γ chain) (clone TUGm2). To perform surface staining, cells were harvested and washed twice with cold 0.5% BSA-containing PBS (FACS buffer). To block the Fc receptor, the cells were incubated with anti-CD16/CD32 mAbs on ice for 10 min and subsequently stained with fluorescently labeled mAbs. Flow cytometric data were acquired using a FACSCalibur flow cytometer (Becton Dickson, San Jose, CA, USA) and analyzed using FlowJo software (version 8.7; Tree Star, Ashland, OR, USA) [37,38].

### 4.4. Intracellular Cytokine Staining

For intracellular staining, splenocytes were incubated with brefeldin A, an intracellular protein transport inhibitor (10 μg/mL), in RPMI medium (Gibco BRL, Gaithersburg, MD, USA) for 2 h at 37 °C. The cells were stained for cell surface markers, fixed with 1% paraformaldehyde, washed once with cold FACS buffer, and permeabilized with 0.5% saponin. The permeabilized cells were then stained for an additional 30 min at room temperature with the indicated mAbs (PE-conjugated anti-TNFα, anti-HIF1α, anti-IL15, or PE-conjugated isotype control rat IgG). More than 5000 cells per sample were acquired using a FACSCalibur, and the data were analyzed using the FlowJo software package (version 8.7; Tree Star, Ashland, OR, USA) [39].

### 4.5. Phosphoflow Analysis of Protein Phosphorylation Levels

Cells were fixed in prewarmed Fix Buffer I (BD Phosflow^TM^ Cat. No. 557870) for 10 min at 37 °C. Immediately after washing with cold PBS, permeabilization was performed with cold PhosflowPermBuffer II (BD Phosflow^TM^ Cat. No. 558050) for 30 min on ice. Next, the cells were washed twice with staining buffer (1× PBS with 2% FBS) for 10 min and subsequently stained with PE-conjugated anti-Phospho-mTOR (Ser2448) mAb in staining buffer for 30 min at room temperature (RT). More than 5000 cells per sample were acquired using the FACSCalibur and analyzed with the FlowJo software package (version 8.7; Tree Star, Ashland, OR, USA) [40].

### 4.6. Statistical Analysis

Statistical significance was determined using Excel (Microsoft, Redmond, WA, USA). Student’s *t*-test was performed for the comparison of two groups (* *p* < 0.05, ** *p* < 0.01, and *** *p* < 0.001 were considered significant in the Student’s *t*-test).

## Data Availability

The data will be available from the corresponding author upon reasonable request.
